# Glutathione supplementation suppresses muscle fatigue induced by prolonged exercise via improved aerobic metabolism

**DOI:** 10.1186/s12970-015-0067-x

**Published:** 2015-02-06

**Authors:** Wataru Aoi, Yumi Ogaya, Maki Takami, Toru Konishi, Yusuke Sauchi, Eun Young Park, Sayori Wada, Kenji Sato, Akane Higashi

**Affiliations:** Laboratory of Health Science, Graduate School of Life and Environmental Sciences, Kyoto Prefectural University, 1-5 Hangi-cho Shimogamo, Sakyo-ku, Kyoto 606-8522 Japan; KOHJIN Life Sciences Company, Ltd., Tokyo, Japan; Laboratory of Food Science, Graduate School of Life and Environmental Sciences, Kyoto Prefectural University, Kyoto, Japan; Division of Applied Biosciences, Graduate School of Agriculture, Kyoto University, Kyoto, Japan

**Keywords:** Glutathione, Dkeletal muscle, Lipid metabolism, Running exercise, PGC-1α

## Abstract

**Backgrounds:**

Glutathione is an endogenous redox couple in animal cells and plays important roles in antioxidant defense and detoxification, although it is unknown if oral glutathione supplementation affects exercise-induced physiological changes. The present study investigated the effect of glutathione intake on exercise-induced muscle metabolism and fatigue in mice and humans.

**Methods:**

ICR mice were divided into 4 groups: sedentary control, sedentary supplemented with glutathione (2.0%, 5 μL/g body weight), exercise control, and exercise supplemented with glutathione. After 2 weeks, the exercise groups ran on a treadmill at 25 m/min for 30 min. Immediately post-exercise, intermuscular pH was measured, and hind limb muscle and blood samples were collected to measure biochemical parameters. In a double-blind, cross-over study, 8 healthy men (35.9 ± 2.0 y) were administered either glutathione (1 g/d) or placebo for 2 weeks. Then, they exercised on a cycle ergometer at 40% maximal heart rate for 60 min. Psychological state and blood biochemical parameters were examined after exercise.

**Results:**

In the mouse experiment, post-exercise plasma non-esterified fatty acids were significantly lower in the exercise supplemented with glutathione group (820 ± 44 mEq/L) compared with the exercise control group (1152 ± 61 mEq/L). Intermuscular pH decreased with exercise (7.17 ± 0.01); however, this reduction was prevented by glutathione supplementation (7.23 ± 0.02). The peroxisome proliferator-activated receptor-γ coactivator-1α protein and mitochondrial DNA levels were significantly higher in the sedentary supplemented with glutathione group compared with the sedentary control group (25% and 53% higher, respectively). In the human study, the elevation of blood lactate was suppressed by glutathione intake (placebo, 3.4 ± 1.1 mM; glutathione, 2.9 ± 0.6 mM). Fatigue-related psychological factors were significantly decreased in the glutathione trial compared with the placebo trial.

**Conclusions:**

These results suggest that glutathione supplementation improved lipid metabolism and acidification in skeletal muscles during exercise, leading to less muscle fatigue.

**Electronic supplementary material:**

The online version of this article (doi:10.1186/s12970-015-0067-x) contains supplementary material, which is available to authorized users.

## Background

Glutathione, a tripeptide consisting of glutamate, cysteine, and glycine, is synthesized primarily in the hepatic cells. It is stored in an oxidized or reduced form at high concentrations in the majority of cells. Glutathione is reported to be involved in the regulation of various physiological functions, in particular, antioxidation and detoxification functions [1,2]. Reduced glutathione is easily oxidized by reactive oxygen species and subsequently reduced again by glutathione reductase, and the redox balance of glutathione has been used as a marker of antioxidant status in various conditions. Physical exercise decreases the reduced form and increases the oxidized form of glutathione [3,4]. In addition, prolonged exercise decreases total plasma and tissue glutathione content over time [5,6], which suggests that glutathione may be associated with aerobic energy metabolism and maintenance of muscle contraction.

Energy and nutrient metabolism in skeletal muscle plays an important role in muscular fatigue. In particular, the energy source can affect muscle performance. For example, a carbohydrate-based energy source results in decreased muscular pH owing to increased lactic acid production, which leads to impaired muscle contraction. However, when the energy expended during exercise is derived from lipids, a large amount of energy can be continuously supplied via aerobic metabolism. Therefore, increased lipid utilization in the mitochondria of skeletal muscle cells is associated with continued muscle contraction, and the number and activity of mitochondria influence the utilization of fatty acids in muscle cells.

Over the past decade, peroxisome proliferator-activated receptor-γ coactivator-1α (PGC-1α) has been demonstrated as a key transcriptional co-activator, providing a mechanistic insight into nuclear regulatory pathways in the biogenesis of mitochondria in skeletal muscle [7-9]. Its contents are changed by differing levels of physical activity, metabolic disorders, and aging, which are all associated with metabolic capacity [9-11]. Moreover, it interacts with nuclear receptors and transcription factors to activate the transcription of lipid metabolic genes, and its activity is responsive to aerobic metabolic events [9,10].

Recently, Kovacs-Nolan et al. [12] demonstrated, in *in vitro* and *ex vivo* intestinal absorption model, that intact glutathione can be transported across human intestinal epithelial cells. In addition, we reported that the plasma glutathione concentration is transiently elevated after oral glutathione supplementation [13], which suggests that exogenous glutathione can be absorbed into the body and act as an important glutathione supply. Therefore, we hypothesized that glutathione supplementation may contribute to aerobic metabolism during exercise as a result of activated mitochondria via PGC-1α in skeletal muscle. The purpose of this study was to examine the effects of glutathione supplementation on muscle fatigue in exercise as a result of improved muscular aerobic metabolism.

## Methods

### Animal experimental design

This study complied with the guidelines of the Japanese Council on Animal Care and was approved by the Committee for Animal Research at Kyoto Prefectural University of Medicine (No. 23–77). ICR mice (7-week-old; Shimizu Laboratory Supplies, Kyoto, Japan) were acclimatized for 1 week in an air-conditioned (22 ± 2°C) room on a 12-h light/dark cycle (lights on from 7:30 to 19:30). Mice were divided into 4 groups containing 8 animals each: sedentary control, sedentary supplemented with glutathione, exercise control, and exercise supplemented with glutathione. Glutathione was purified and crystalized after separation from the fermented food-grade yeast *Candida utilis* which approved by the U.S. food and drug administration (FDA), and used for experiments.

A glutathione solution (2% w/w) was provided 1 time per d (5 μL/g body weight) for 2 weeks. Saline was provided to control mice in the same volume. After 2 weeks of treatment, the exercise groups ran on a treadmill; after an initial 5-min warm up, the running speed was gradually increased to 25 m/min, which was maintained for 30 min. Immediately following the exercise, intermuscular pH was measured under anesthesia; then the hind limb muscles and blood were collected. The control mice underwent the same measurements. Blood glucose was measured (GluTest; Arkray, Inc., Kyoto, Japan), following which the blood samples were centrifuged at 3,500 rpm for 15 min at 4°C to collect plasma. Plasma nonesterified fatty acid (NEFA) was measured using a NEFA-C assay kit (WAKO, Osaka, Japan).

### pH measurements in animals

pH levels in the interstitial fluid of exercised muscle tissue were measured under anesthesia using a glass microelectrode that was inserted into the interstitium between the gastrocnemius and tibialis anterior muscles.

### Western blotting

Protein was extracted from muscle tissues obtained from sedentary groups using a lysis buffer (Sigma, St. Louise, MO) containing protease inhibitor. Equal amounts of protein in the lysates were separated by 10% sodium dodecyl sulfate-polyacrylamide gel electrophoresis, and proteins were then transferred onto nitrocellulose membrane. The blots were incubated with primary antibodies against PGC-1α (Chemicon International, Temecula, CA) and AMP-activated protein kinase (AMPK) (Cell Signaling Technology, Beverly, MA), and the reaction products were visualized using a horseradish peroxidase-conjugated secondary antibody (Invitrogen, Carlsbad, CA) and enhanced chemiluminescence (Chemi-Lumi One Super, Nakarai Tesque, Kyoto, Japan) (Additional file 1: Figure S1). Band densities were measured using ImageQuant LAS4000 (GE Healthcare, Buckinghamshire, UK).

### Mitochondrial DNA

To determine the mitochondrial DNA (mtDNA), polymerase chain reaction (PCR) analysis was performed using DNA obtained from muscle tissues. Total DNA was extracted using the extraction reagent (DNA zol® BD Reagent, Invitrogen) following homogenization. The relative copy numbers of mitochondrial to nuclear DNA were determined by real-time PCR with primers specific to cytochrome c oxidase subunit II (COX II) (mitochondrial) 5′-ATCCCAGGCCGACTAAATCA (forward) and 5′-TTTCAGAGCATTGGCCATAGAA (reverse) and β-actin (nuclear) genes 5′-TATCCACCTTCCAGCAGATGT (forward) and 5′-AGCTCAGTAACAGTCCGCCTA (reverse).

### Human experimental design

Eight healthy men (age, 35.9 ± 2.0 y; height, 172.6 ± 1.9 cm; body weight, 70.6 ± 3.2 kg; BMI, 23.8 ± 1.2 kg/m^2^) were recruited to participate in a double-blind, cross-over study, which was complied with the principles outlined in the Helsinki Declaration and approved by the ethics committee of Kyoto Prefectural University (No. 46). All of the subjects provided written, informed consent. The subjects did not have a current or prior chronic disease or a history of smoking, and they were not currently using any medications. The subjects were also not habituated to a regular exercise regimen.

A single-bout exercise experiment was performed following 2 weeks of glutathione (1 g/d) or placebo supplementation in capsule form. The subjects were asked to fast, except for water, from 22:00 the night prior to the experiment. On the experiment day, all subjects consumed the same breakfast (200 g of steamed rice) 90 min before the exercise session to normalize the effects of a pre-exercise meal. All participants performed a single bout of steady-state cycling exercise at 40% maximum heart rate for 60 min. Heart rate and the rating of perceived exertion (RPE; Borg scale) were monitored every 10 min during exercise. Blood samples were collected from the antecubital vein before and after exercise, and blood glucose and lactate were measured using simple measuring instruments (Lactate Pro, GluTest; Arkray, Kyoto, Japan). Data obtained from seven subjects was analyzed for glucose and lactate due to failure of blood collection during exercise for one subject. Psychological state was assessed after blood collection using the Profile of Mood State test, of which we were interested in 2 mood factors (fatigue–inertia and vigor–activity). In addition, the degree of relaxation was examined using a visual analogue scale (VAS). A 4-week washout period separated the conditions, and all of the subjects completed both testing conditions.

### Measurement of plasma glutathione

Plasma glutathione concentration was measured according to our previously reported protocol [13]. Briefly, collected plasma was mixed with ethanol and then centrifuged. The supernatant was dried and added to 5% trichloroacetic acid (TCA) containing 2% 2-mercaptoethanol to reduce the oxidized form of glutathione, and the mixture was used as the deprotenized plasma fraction. The ethanol precipitate of the plasma was mixed with the 5% (final concentration) TCA and 2% 2-mercaptoethanol. After centrifugation, the supernatant was used as the protein-bound plasma fraction. The concentrations of both fractions were determined by precolumn derivation with 6-aminoquinolyl-N-hydroxy succinimidyl carbamate and liquid chromatography-tandem mass spectrometry (LC-MS/MS).

### Statistical analysis

All data are reported as mean ± SE. Differences between groups or time course comparisons were evaluated using 2-way ANOVA. When significant interactions were detected, *post hoc* multiple comparisons were conducted using the Bonferroni method. Differences between the control and glutathione groups in the animal study were determined using Student’s *t*-tests. Differences between the placebo and glutathione trials in the human study were tested using Wilcoxon signed-rank tests. P < 0.05 was considered statistically significant.

## Results

### Body weight and blood parameters in animals

**Figure 1:**
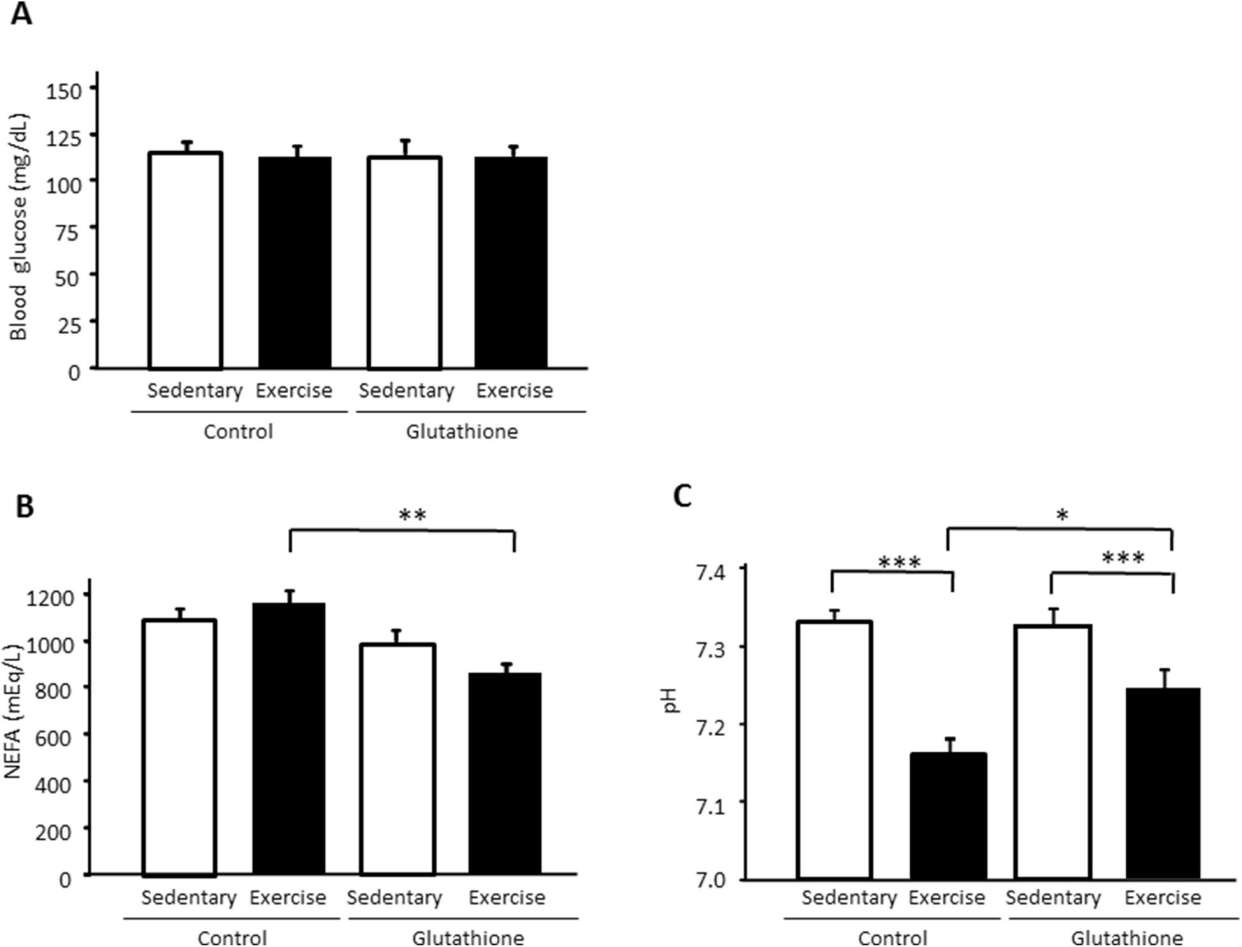
**The effect of glutathione on blood glucose (A), NEFA (B), and intermuscular pH (C) in sedentary and exercised mice.** Values are provided as mean ± SE. *p < 0.05, **p < 0.01, ***p < 0.001.

Body weight was similar in all of the groups (sedentary control, 41.4 ± 0.9 g; sedentary supplemented with glutathione, 39.8 ± 0.9 g; exercise control 41.3 ± 0.8 g; exercise supplemented with glutathione, 40.2 ± 0.7 g). There were no significant differences in blood glucose levels between the control and glutathione groups in both sedentary and exercise conditions (degree of freedom (df) = 31, F = 0.039) (Figure 1A). In contrast, plasma NEFA after exercise was significantly lower with supplementation of glutathione compared with the control group (df = 31, F = 5.90, p < 0.01) (Figure 1B).

### Intermuscular pH in animals

The interstitial pH levels in muscle were significantly reduced by exercise (df = 31, F = 4.36, p < 0.001; Figure 1C). However, the pH following exercise of the glutathione group was significantly higher than that of the control group (p < 0.05).

### PGC-1α, AMPK, and mitochondrial DNA in mouse muscle

**Figure 2:**
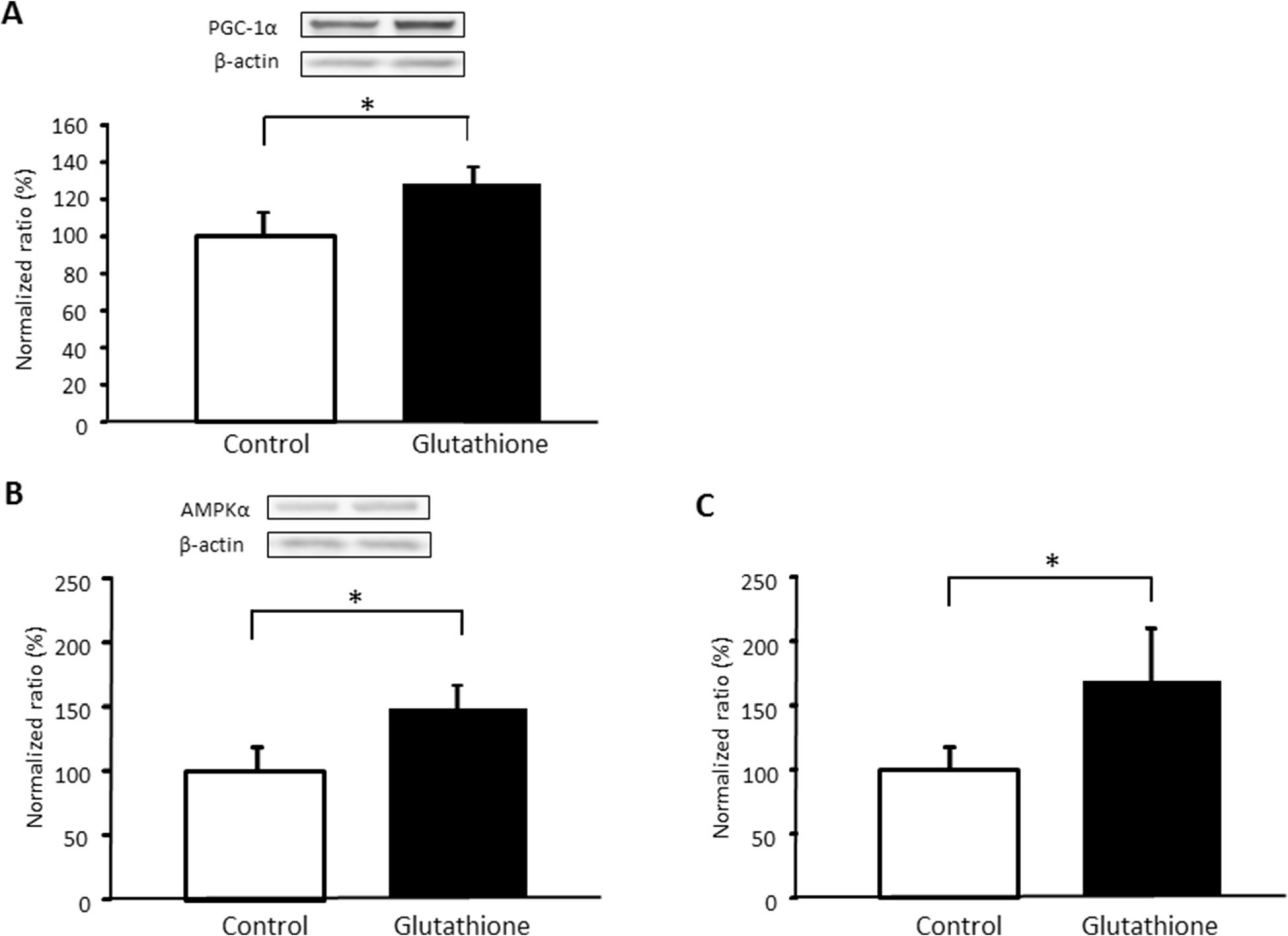
**The effect of glutathione on PGC-1α (A), AMPK (B) and mtDNA (C) levels in skeletal muscle in sedentary mice.** Values are provided as mean ± SE. *p < 0.05.

PGC-1α was significantly higher with glutathione intake (df = 14, t = −1.88, p < 0.05; Figure 2A). In addition, AMPK, an upstream protein of PGC-1α, was also significantly higher in the sedentary treated with glutathione group than in the sedentary control group (df = 13, t = −2.76, p < 0.05; Figure 2B). In addition, mtDNA was significantly higher with glutathione supplementation (df = 14, t = −1.98, p < 0.05; Figure 2C).

### Blood biochemical parameters in humans

**Figure 3:**
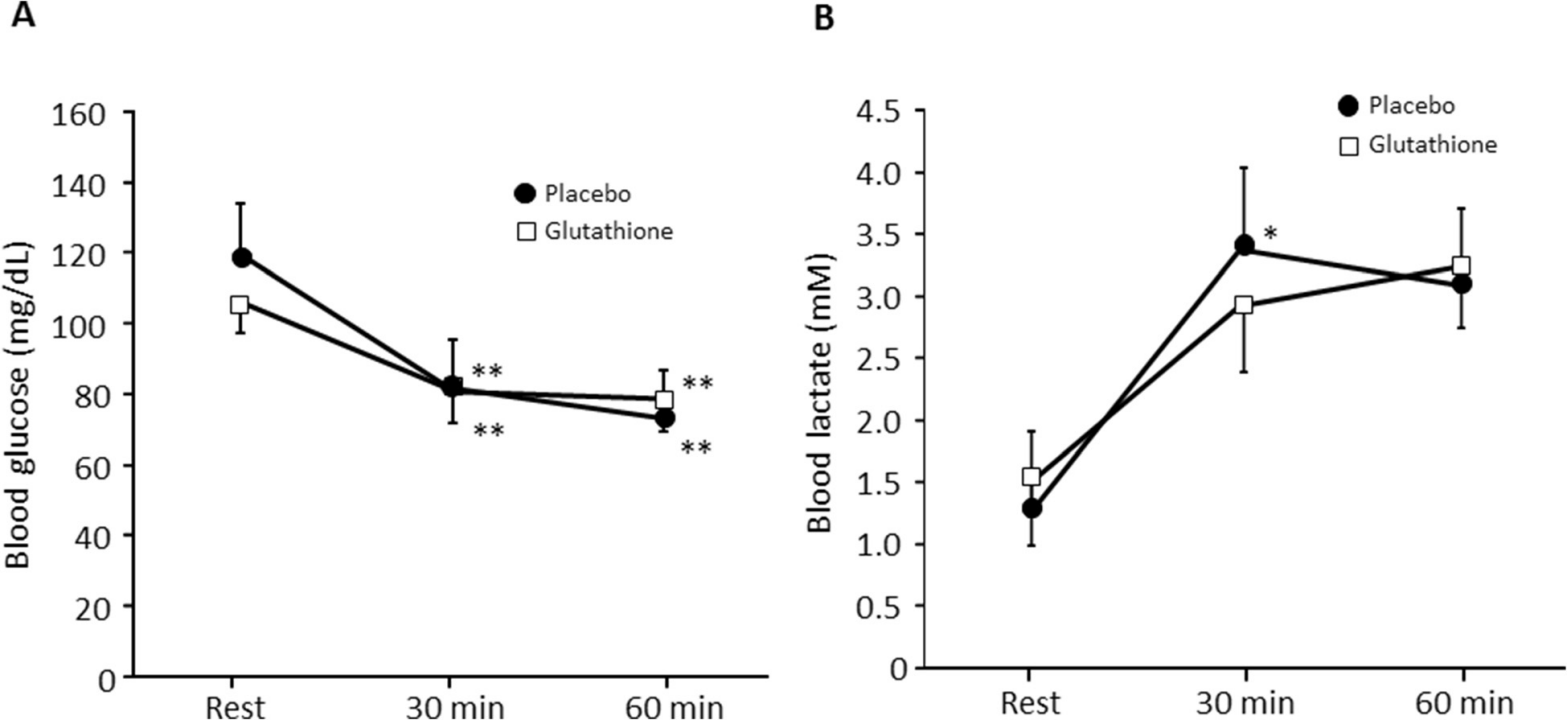
**The effect of glutathione on blood glucose (A) and lactate (B) level in humans.** Values are provided as the mean ± SE. *p < 0.05, **p < 0.01 vs. Rest.

**Figure 4:**
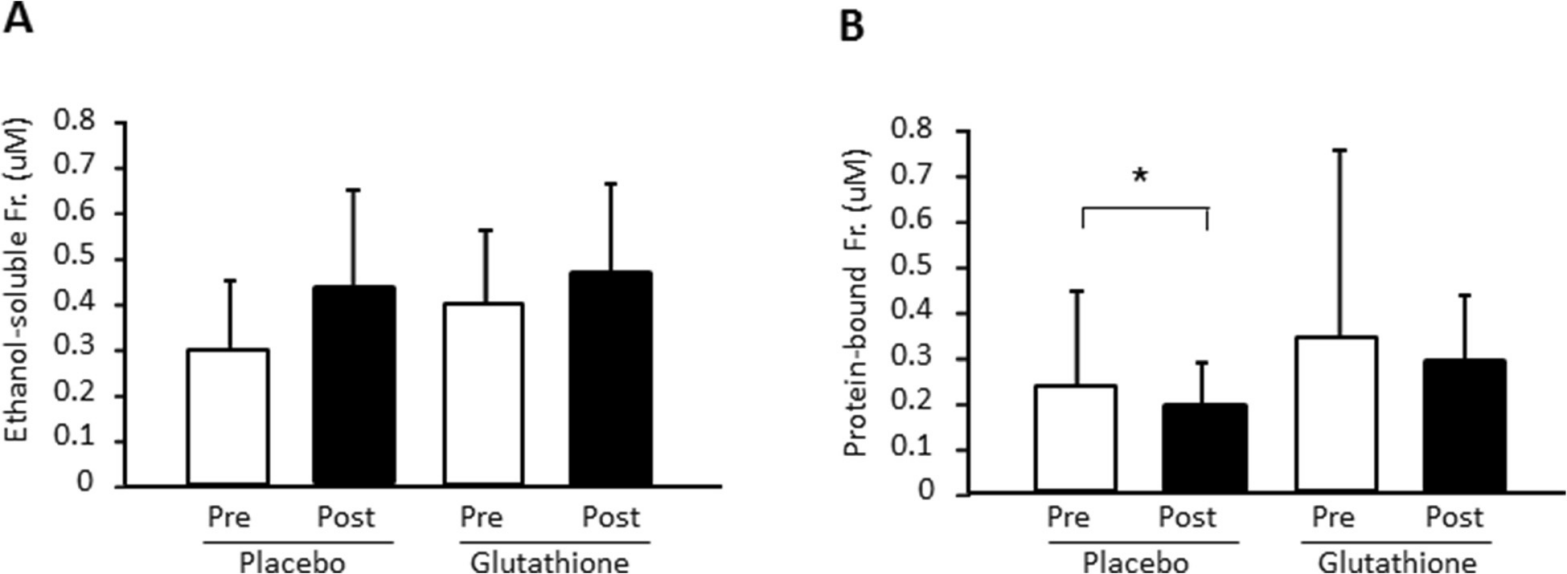
**The effect of glutathione on plasma ethanol-soluble (A) and protein-bound (B) glutathione level in humans.** Values are provided as the mean ± SE. *p < 0.05.

There was a significant decrease in the blood glucose concentrations at 30 and 60 min after exercise compared with pre-exercise in both the placebo and glutathione trials (df = 41, F = 23.9, p < 0.01) (Figure 3A); however, there was no difference between the trials. There was a significant increase in blood lactate concentrations at 30 min after exercise compared with pre-exercise in the placebo trial (df = 41, F = 3.90, p < 0.05) but not in the glutathione trial (Figure 3B). The free form of glutathione in plasma was not changed by either exercise or glutathione intake. In contrast, protein-bound plasma glutathione was significantly reduced by exercise in the placebo trial (p < 0.05) although the reduction was moderated in the glutathione-supplemented group (Figure 4).

### Heart rate and psychological parameters in humans

**Figure 5:**
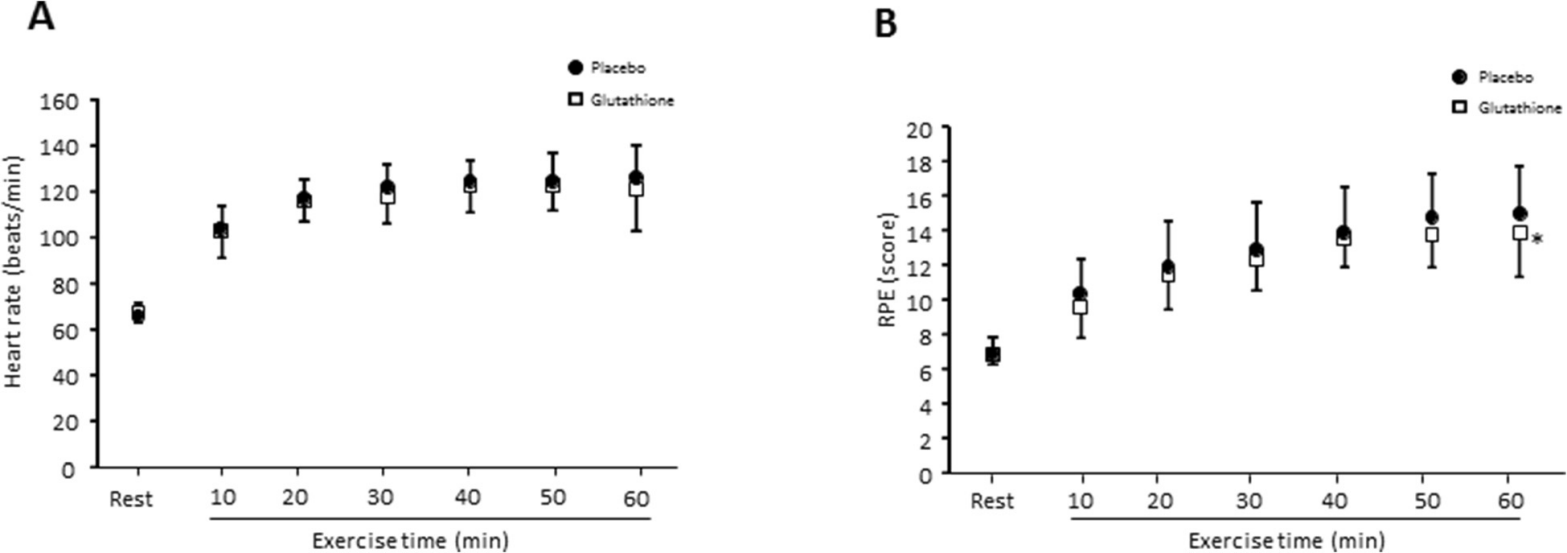
**The effect of glutathione on heart rate (A) and the rating of perceived exertion (B) level in in humans.** Values are provided as the mean ± SE. *p < 0.05 vs. placebo.

There was a trend for lower heart rates during exercise at 40 and 60 min in the glutathione trial compared with the placebo trial (Z = −1.47, p = 0.071 and Z = −1.26, p = 0.104, respectively) (Figure 5). There was also a trend for a lower RPE at 50 min (Z = −1.44, p = 0.075) and a significant decrease at 60 min (Z = −1.78, p < 0.05) in the glutathione trial compared with the placebo trial.

The Profile of Mood State vigor–activity factor after exercise was significantly higher following exercise in the glutathione trial compared with the placebo trial (Z = −2.11, p < 0.05) (Table 1). In contrast, the fatigue–inertia factor was significantly lower in the glutathione trial compared with the placebo trial (Z = −1.82, p < 0.05), while marked difference was not found in the VAS scores between trials (Z = −0.98, p = 0.163).

**Table 1:** **Psychological examination after exercise**

	**Placebo**	**Glutathione**
**POMS test**		
Vigor-activity	54.5 ± 2.8	58.8 ± 3.2*
Fatigue-inertia	51.0 ± 2.8	45.1 ± 2.7*
**VAS**	6.0 ± 0.7	4.7 ± 0.8

Values are provided as mean ± SE. *p < 0.05 vs. placebo.

POMS, Profile of Mood State; VAS, visual analogue scale.

## Discussion

In the present study, glutathione supplementation resulted in higher levels of PGC-1α and mtDNA biogenesis in mouse skeletal muscle and prevented the exercise-induced reduction in intermuscular pH in mice. Moreover, in humans, glutathione supplementation suppressed fatigue-related parameters during and after exercise. While it has been well documented that glutathione plays a central role in the antioxidant network in animal cells and redox balance can act as a marker of antioxidant status in various pathological and physiological conditions including exercise [3,4,14,15], the role of exogenous glutathione on phenotypic changes relating to physical exercise has not been explored. To the best of our knowledge, the present study is the first to demonstrate that glutathione supplementation improves aerobic metabolism in skeletal muscle, leading to reduced exercise-induced muscle fatigue.

During muscle contraction, lactic acid, a major source of protons, is rapidly produced by increased glycolytic metabolism, lowering the pH and inhibiting muscle contraction [16]. The protons generated from cytosolic lactic acid are immediately buffered in the cell or exported to the interstitial fluid and further transported to the blood. The buffering capacity is relatively high in the cytosol and blood, whereas this capacity is low in interstitial fluid where the presence of buffering factors, such as proteins, is limited [17,18]. Therefore, the pH of the interstitial fluid in muscle tissues can drastically change in response to muscle contraction and can also be a marker of acid–base conditions in muscle tissue. In contrast, the majority of lactate anions are released into the circulation or immediately metabolized as an energy substrate through aerobic metabolism [19-21]; therefore, their levels are not suitable as a marker. The results of both the animal and human studies indicate that glutathione supplementation inhibited the decrease in intermuscular pH after exercise; in the human study, this was demonstrated by the differences in blood lactate concentrations following exercise between the placebo and glutathione trials. These results may also explain the differences observed between the trials in RPE and subjective fatigue during and after exercise; in particular, an improvement in muscular acidosis results in less fatigue.

Circulating NEFA concentrations are regulated by a balance between catabolic processes in adipose tissue and fatty acid substrate utilization by the skeletal muscle. Circulating catecholamines such as adrenalin and noradrenalin are increased in response to exercise and stimulate lipolysis of triglycerides in adipose tissue [22], which causes an elevation of circulating fatty acids. In contrast, muscle contraction increases uptake of fatty acids from the circulation into muscle cells [23], which leads to a decrease in circulating fatty acids. Therefore, we suggest that the reduction of NEFA observed in the glutathione-supplemented mice was due to an increase in muscle utilization rather than a release from adipose tissue. Because energy consumed in muscle during exercise is mainly supplied by carbohydrates and lipids, glutathione-induced lipid utilization can decrease energy obtained from carbohydrates, which may lead to a decrease in lactate/proton production. Collectively, this indicates that glutathione improves metabolic acidosis through the activation of lipid metabolism, which leads to suppression of exercise-induced fatigue.

PGC-1α is a central member of a family of transcriptional co-activators involved in aerobic metabolism. Activation of PGC-1α alters the metabolic phenotype through interactions with nuclear respiratory factor and peroxisome proliferator-activated receptor-α [8-10], which leads to increased mitochondrial biogenesis and activity. It has been reported that PGC-1α activation causes significant improvements in athletic performance [24,25], prevention and treatment of muscle weakness in the elderly, obesity, and other metabolic diseases such as mitochondrial myopathies and diabetes [10,11,26]. Here, we detected an increase in PGC-1α with 2 weeks of glutathione intake, along with an increase in mtDNA content, indicating the activation of mitochondrial biogenesis. Therefore, the observed elevation of PGC-1α by glutathione intake strongly suggests an acceleration in lipid metabolism. In addition, the increase of mitochondria content could also lead to a decrease of lactate generation by accelerating aerobic metabolism of glucose, which would prevent muscle acidosis during exercise even further.

The regulatory mechanism for glutathione-induced increases in PGC-1α is unclear. One explanation is the elevation of AMPK, which is an upstream factor of PGC-1α regulation [27,28]. Recently, it has been argued that oral intake of other antioxidants, including vitamin C and E, do not elevate PGC-1α in the skeletal muscle of mice and humans [29,30]; thus, this may be a specific action of glutathione as a signal factor, but not its antioxidant properties. We found that 2 weeks of glutathione supplementation did not affect plasma glutathione concentration in the basal state. However, glutathione is transported across the intestines with in its intact form [12], and its plasma concentration, along with the glutathione-derived dipeptides γ-glutamyl-cysteine and cysteinyl-glycine, is markedly elevated during the 60–120-min period after oral administration, as shown in our previous report [13]. Therefore, the transient elevation of glutathione or the derived dipeptides following supplementation over 2 weeks may indicate stimulation of specific signaling factors that lead to elevated AMPK and PGC-1α. Alternatively, glutathione content in muscle tissues may also increase with supplementation, leading to the up-regulation of these factors. Further studies are needed to determine the specific mechanism(s) by which glutathione affects muscle aerobic metabolism. In addition, we also observed that reduction of the protein-bound glutathione concentration in plasma after exercise was suppressed following glutathione supplementation, which may also be related to the regulation of energy metabolism or fatigue. Future studies should also aim to identify the bound protein in plasma and examine the mechanism of protein binding or release from the protein and its source.

## Conclusions

The present results demonstrated that 2 weeks of glutathione supplementation decreased plasma fatty acids and suppressed the exercise-induced reduction in intermuscular pH. Glutathione supplementation also resulted in elevated concentrations of PGC-1α and mitochondria in skeletal muscle. These observations suggest that glutathione induces aerobic metabolism and improves an acidic environment in skeletal muscle during exercise by elevating PGC-1α, which would prevent exercise-induced fatigue.

## Additional file

Additional file 1: Figure S1. Images of western blotting for PGC-1α, AMPK, and β-actin in control (C) and glutathione (G) groups.

## Abbreviations

AMPK: AMP-activated protein kinase
COX II: Cytochrome c oxidase subunit II
mtDNA: Mitochondrial DNA
NEFA: Nonesterified fatty acid
PCR: Polymerase chain reaction
PGC-1α: Peroxisome proliferator-activated receptor-γ coactivator-1α
RPE: Rating of perceived exertion
VAS: visual analogue scale
