# Supplementary material for: Glutathione supplementation suppresses muscle fatigue induced by prolonged exercise via improved aerobic metabolism
Source: J Int Soc Sports Nutr. 2015 Feb 6;12:7. doi: 10.1186/s12970-015-0067-x (PMC4328900; doi:10.1186/s12970-015-0067-x)
Supplement: Additional file 1: Figure S1. — Images of western blotting for PGC-1α, AMPK, and β-actin in control (C) and glutathione (G) groups. [file 12970_2015_67_MOESM1_ESM.pdf]

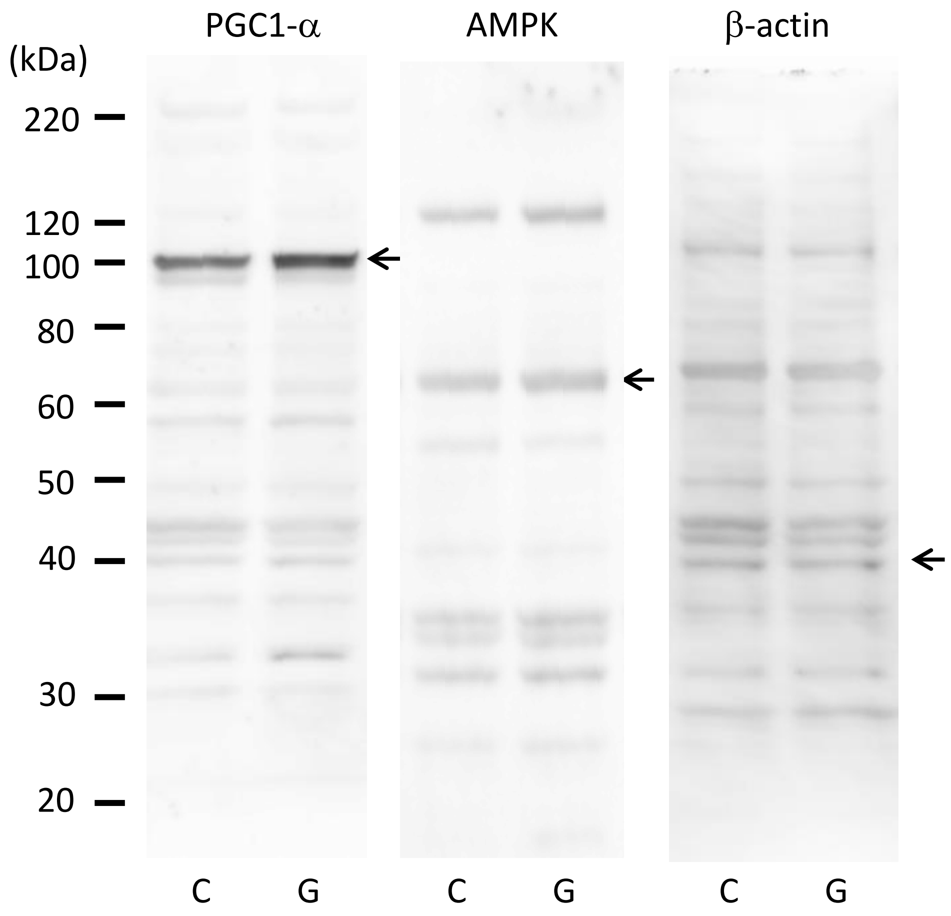


Supplementary figure 1. Images of western blotting for PGC-1α, AMPK, and β-actin in control (C) and glutathione (G) groups.
